# Comparison of Bootstrap Confidence Interval Methods for GSCA Using a Monte Carlo Simulation

**DOI:** 10.3389/fpsyg.2019.02215

**Published:** 2019-10-11

**Authors:** Kwanghee Jung, Jaehoon Lee, Vibhuti Gupta, Gyeongcheol Cho

**Affiliations:** ^1^Department of Educational Psychology and Leadership, Texas Tech University, Lubbock, TX, United States; ^2^Department of Computer Science, Texas Tech University, Lubbock, TX, United States; ^3^Department of Psychology, McGill University, Montreal, QC, Canada

**Keywords:** structural equation modeling (SEM), bootstrap methods, generalized structured component analysis (GSCA), confidence intervals, Monte Carlo simulation

## Abstract

Generalized structured component analysis (GSCA) is a theoretically well-founded approach to component-based structural equation modeling (SEM). This approach utilizes the bootstrap method to estimate the confidence intervals of its parameter estimates without recourse to distributional assumptions, such as multivariate normality. It currently provides the bootstrap percentile confidence intervals only. Recently, the potential usefulness of the bias-corrected and accelerated bootstrap (BCa) confidence intervals (CIs) over the percentile method has attracted attention for another component-based SEM approach—partial least squares path modeling. Thus, in this study, we implemented the BCa CI method into GSCA and conducted a rigorous simulation to evaluate the performance of three bootstrap CI methods, including percentile, BCa, and Student's *t* methods, in terms of coverage and balance. We found that the percentile method produced CIs closer to the desired level of coverage than the other methods, while the BCa method was less prone to imbalance than the other two methods. Study findings and implications are discussed, as well as limitations and directions for future research.

## Introduction

Generalized structured component analysis (GSCA; Hwang and Takane, 2004, 2014) is an approach to component-based structure equation modeling (SEM), where constructs are represented by weighted composites or components of indicators (observed variables; Tenenhaus, 2008; Rigdon, 2012). Since Hwang and Takane (2004) seminal work, the data-analytic capability of GSCA has been markedly improved allowing for applied researchers, for example, to handle cluster-level respondent heterogeneity (Hwang et al., 2007a), multilevel modeling (Hwang et al., 2007b; Jung et al., 2016), moderating effects of constructs (Hwang et al., 2010), and longitudinal data and time series/functional data (Jung et al., 2012, 2016; Suk and Hwang, 2016).

GSCA uses an alternating least squares (De Leeuw et al., 1976) algorithm to minimize a single least squares function for parameter estimation without requiring distributional assumptions such as multivariate normality. Thus, it is a distribution-free approach. As a trade-off, however, it cannot estimate the standard errors or confidence intervals of its parameter estimates based on asymptotic (normal theory) approximations (Hwang and Takane, 2014, pp. 24–25). Instead, it utilizes the bootstrap method (Efron, 1982) to obtain the standard errors and confidence intervals non-parametrically. In the first step, random samples of size *n* (equal to the size of the original data set) are repeatedly sampled from the original data set with replacement. In the second step, the parameters are estimated using each bootstrap sample. Lastly, the standard errors and confidence intervals are derived from the relative frequency distribution of the estimates over the resamples considered as an empirical approximation of its sampling distribution. The bootstrap standard errors and confidence intervals can be used to test statistical significance of the parameter estimates. For example, a bootstrap *t* statistic, also called critical ratio (CR), can be calculated by dividing a parameter estimate by its bootstrap standard error. If the bootstrap *t* value is equal to or greater than the critical value of a *t* distribution, the parameter estimate is considered statistically significant at the nominal alpha level under the assumption that the empirical sampling distribution of the parameter is approximately *t*-distributed. GSCA currently provides percentile confidence intervals (Kim et al., 2017; Hwang et al., 2019). A confidence interval (CI) is defined as the interval between the lower and upper bounds of a parameter estimate at a prespecified confidence level (e.g., 95% confidence interval). The use of percentile CIs is recommended over critical ratios because the percentile CIs deliver more information about the properties of parameter estimates, including precision, and statistical significance without the normality assumption of the estimates (Efron, 1982).

Recently, bias-corrected and accelerated bootstrap (BCa) CI has been suggested for its use with partial least square path modeling (Hair et al., 2016, pp. 155–159), another approach to component-based SEM (Wold, 1982). More recently, Aguirre-Urreta and Rönkkö (2018) conducted a rigorous simulation study and showed that, overall, the percentile method tended to produce more conservative CIs—overcoverage (i.e., wider CIs), whereas the BCa method tended to provide too narrow CIs—undercoverage (i.e., narrower CIs). Nevertheless, this study recommended the use of the percentile method over the BCa method for partial least square path modeling because the population value was not often enough covered by BCa CIs. No study has yet investigated the performance of the bootstrap CI methods for GSCA. Thus, in this study, we implement the BCa CI method into GSCA and conduct a rigorous simulation to examine the performance of different bootstrap CI methods for GSCA, including the percentile, BCa, and Student's *t* methods.

The organization of the article is as follows: we begin by providing a description of the different bootstrap CI methods. We then discuss the design and analysis procedure of our Monte Carlo simulation study and report its results. The final section summarizes the findings and implications of the study as well as discusses its limitations and directions for future research.

## Bootstrap Confidence Interval Methods

As stated, we focus on the three bootstrap CI methods that are most popular in practice: percentile, bias-corrected and accelerated CI, and Student's *t* (Efron and Tibshirani, 1993; Chernick, 2011).

### Percentile Bootstrap Method

The percentile bootstrap interval is just the interval between the $100\times \left(\frac{\alpha }{2}\right)$ and $100\times \left(1-\frac{\alpha }{2}\right)$ percentiles of the distribution of θ estimates obtained from resampling, where θ represents a parameter of interest and α is the level of significance (e.g., α = 0.05 for 95% CIs) (Efron, 1982). A bootstrap percentile CI of $\hat{\theta }$ (an estimator of θ) can be obtained as follows: (1) *B* random bootstrap samples are generated, (2) a parameter estimate is calculated from each bootstrap sample, (3) all B bootstrap parameter estimates are ordered from the lowest to highest, and (4) the CI is constructed as follows,

$$\begin{array}{l} \left[{\hat{\theta }}_{\text{lower limit}},\;{\hat{\theta }}_{\text{upper limit}}\right]=\left[{\hat{\theta }}_{j}^{*},\;{\hat{\theta }}_{k}^{*}\right], \end{array}$$

where ${\hat{\theta }}_{j}^{*}$ denotes the *j*th quantile (lower limit), and ${\hat{\theta }}_{k}^{*}$ denotes the *k*th quantile (upper limit); $j=\left[\frac{\alpha }{2}\times B\right],\;k=\left[\left(1-\frac{\alpha }{2}\right)\times B\right]$. For example, a 95% percentile bootstrap CI with 1,000 bootstrap samples is the interval between the 25th quantile value and the 975th quantile value of the 1,000 bootstrap parameter estimates.

### Bias-Corrected and Accelerated Bootstrap Method

To overcome the overcoverage issues in percentile bootstrap CIs (Efron and Tibshirani, 1993), the BCa method corrects for both bias and skewness of the bootstrap parameter estimates by incorporating a bias-correction factor and an acceleration factor (Efron, 1987; Efron and Tibshirani, 1993). The bias-correction factor ẑ_0_ is estimated as the proportion of the bootstrap estimates less than the original parameter estimate $\hat{\theta }$,

$$\begin{array}{l} {ẑ}_{0}=\Phi^{-1}\left(\frac{\#\left\{{\hat{\theta }}^{*}<\;\hat{\theta }\right\}}{B}\right), \end{array}$$

where Φ^−1^ is the inverse function of a standard normal cumulative distribution function (e.g., Φ^−1^ (0.975) = 1.96). The acceleration factor â is estimated through jackknife resampling (i.e., “leave one out” resampling), which involves generating *n* replicates of the original sample, where *n* is the number of observations in the sample. The first jackknife replicate is obtained by leaving out the first case (*i* = 1) of the original sample, the second by leaving out the second case (*i* = 2), and so on, until *n* samples of size *n*−1 are obtained. For each of the jackknife resamples, ${\hat{\theta }}_{\left(-i\right)}$ is obtained. The average of these estimates is,

$$\begin{array}{l} {\hat{\theta }}_{\left(\cdot \right)}=\sum_{i=1}^{n}\frac{{\hat{\theta }}_{\left(-i\right)}}{n} \end{array}$$

Then, the acceleration factor â is calculated as follows,

$$\begin{array}{l} â=\frac{\sum_{i=1}^{n}{\left({\hat{\theta }}_{\left(\cdot \right)}-{\hat{\theta }}_{\left(-i\right)}\right)}^{3}}{6{\left\{\sum_{i=1}^{n}{\left({\hat{\theta }}_{\left(\cdot \right)}-{\hat{\theta }}_{\left(-i\right)}\right)}^{2}\;\right\}}^{3/2}}. \end{array}$$

With the values of ẑ_0_ and â, the values α_1_and α_2_ are calculated,

$$\begin{array}{l} \alpha_{1}=\Phi \left\{{ẑ}_{0}+\frac{{ẑ}_{0}+\;z^{\left(\alpha /2\right)}}{1-â\left({ẑ}_{0}+\;z^{\left(\alpha /2\right)}\right)}\right\}\\ \alpha_{2}=\Phi \left\{{ẑ}_{0}+\frac{{ẑ}_{0}+\;z^{\left(1-\alpha /2\right)}}{1-â\left({ẑ}_{0}+\;z^{\left(1-\alpha /2\right)}\right)}\right\} \end{array}$$

Here, *z*^(α/2)^ is the $100\times \left(\frac{\alpha }{2}\right)$th percentile point of a standard normal distribution (e.g., *z*^(.05/2)^ = −1.96). Then, a CI $\left[{\hat{\theta }}_{j}^{*},\;{\hat{\theta }}_{k}^{*}\;\right]$ is constructed with the values of α_1_ and α_2_ multiplied by the number of bootstrap samples, *j* = α_1_ × *B* and *k* = α_2_ × *B*.

### Bootstrap Student's *t* Method

Student's *t* method assumes that $\frac{\hat{\theta }-\theta }{\hat{se}}$ is approximately *t*-distributed, where θ is a parameter, $\hat{\theta }$ is an estimate of the parameter, and $\hat{se}$ is the standard error of the parameter. Bootstrap Student's *t* CI for a certain alpha level (e.g., α = 0.05 for 95% CIs) is constructed as follows,

$$\begin{array}{l} \left[\hat{\theta }-t_{n-1}^{\left(\frac{a}{2}\right)}\cdot \hat{se},\;\hat{\theta }+t_{n-1}^{\left(1-\frac{a}{2}\right)}\cdot \hat{se}\;\right]. \end{array}$$

The bootstrap standard error of each estimate, $\hat{se}\left({\hat{\theta }}^{*}\right)$, is used for $\hat{se}$. Note that Student's *t* confidence interval has no feature to adjust the confidence interval, accounting for any other types of deviation from the *t* distribution.

## Simulation Design

**Figure 1:**
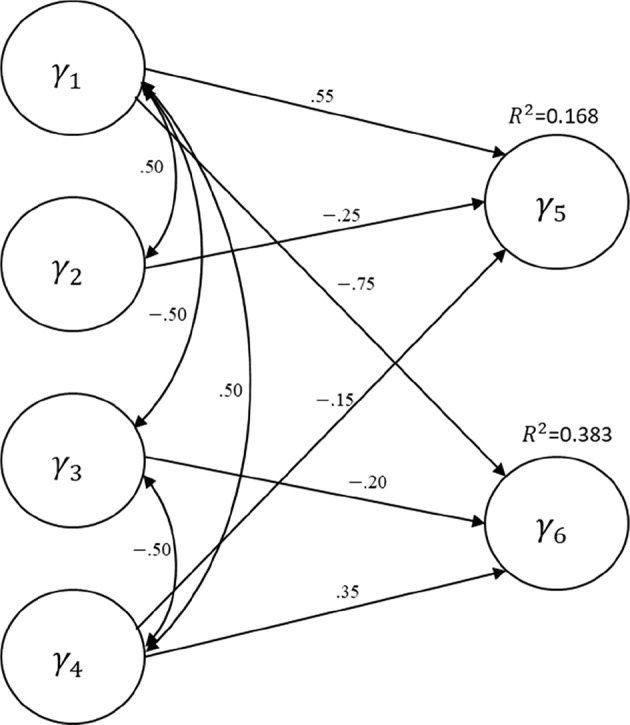
The population structural equation model specified for the simulation study.

We evaluate the performance of the three bootstrap methods (percentile, BCa, and Student's *t*) in GSCA, using a Monte Carlo simulation. Figure 1 depicts the structural layout of the data-generating model (i.e., population model), which was also previously employed in Cho et al. (2019)^1^. The structural model contains four exogenous and two endogenous latent variables^2^, and a few regression paths were specified between them with path coefficient values ranging from −0.75 to 0.55. The variances of the endogenous latent variables explained by the exogenous latent variables (*R*^2^) were 0.168 for γ_5_ and 0.383 for γ_6_. The measurement part of the population model (i.e., measurement model) was homogeneous for the latent variables. That is, each latent variable had three indicators of which standardized loadings were 0.7, 0.8, or 0.9. Note that the symbols for the error terms in the measurement and structural models are omitted in the figure for simplicity.

We considered two different distributions for the indicators—normally distributed with a zero mean and unit variance vs. non-normally distributed through lognormal transformation of the normally distributed indicators. For the non-normally distributed indicators, independent normal random variates were generated, and they were transformed into lognormal random variates by exponentiation, and then these manipulated random variates were standardized. The desired correlation structure was obtained by multiplying the data matrix by the Cholesky factor of the prespecified covariance matrix (Ringle et al., 2014). For the non-normal indicators, the average skewness ranged from 1.41 to 2.53, and the average kurtosis ranged from 6.32 to 18.28. Note that the skewness and kurtosis of a normal distribution are 0 and 3, respectively. We also considered four different sample sizes: *N* = 50, 100, 200, and 500. Five hundred random samples were drawn under each of eight conditions (two distributions × four sample sizes), yielding a total of 4,000 replications. We applied GSCA to fit the model to each sample and, subsequently, obtained the percentile, BCa, and Student's *t* CIs for the loading and path coefficient estimates based on 1,000 bootstrap samples (*B* = 1,000). Specifically, we modified the *gesca* R package (Hwang et al., 2016; R Core Team, 2017) with the new R functions of the bootstrap methods. The MATLAB codes used to generate data and the R functions for three CI calculations are available as Supplementary Materials. See also Cho et al. (2019) and Dijkstra (2017) for more details on the data generation procedure of component-based SEM. There was no case of model non-convergence or inadmissible solution in the current simulation.

## Evaluation Criteria

We evaluated two properties of a CI in the simulation: (a) coverage and (b) balance (Aguirre-Urreta and Rönkkö, 2018). The coverage of a CI is the proportion that the parameter value is included within the CI across replicated samples. Coverage values should be close to a predefined confidence level of the interval. For example, ideally, a 95% CI (a nominal coverage) should include the parameter of interest 95% of the time over replications. In other words, in 5% of the replications, the interval would not capture the “true” parameter value in the population.

The balance of a CI refers to how the “non-coverage” is split. That is, how many times the population value is greater than the upper limit of the interval and how many times the population value is smaller than the lower limit of the interval. In an ideal situation, a CI should be balanced such that the population value is greater than the upper limit or smaller than the lower limit at the same number of times across replications (e.g., 2.5% of times for a 95% CI), while achieving the desired level of coverage.

## Results

This section provides the results of the simulation study, displaying a series of plots that show the performance of the three bootstrap CI methods in terms of coverage and balance averaged over the six latent variables. In these plots, the *x*-axis indicates sample size (*N* = 50, 100, 200, 500) and the *y*-axis the proportion of a loading (0.7, 0.8, 0.9) or path coefficient (−0.75, −0.25, −0.2, −0.15, 0.35, 0.55) being above the upper limit or below the lower limit of a CI. For a 95% CI, this should not occur more than 5%. Furthermore, the parameter value is expected to be above (below) the upper (lower) limit of the CI in a balanced way—i.e., no more than 2.5% in each case. The horizontal dashed line indicates the theoretical coverage range of a perfectly balanced 95% CI. Thus, the closer the line representing a bootstrap CI method to the dashed lines, the closer the particular method is to the ideal coverage and balance that the CI should exhibit.

### Loadings for Normally Distributed Indicators

**Figure 2:**
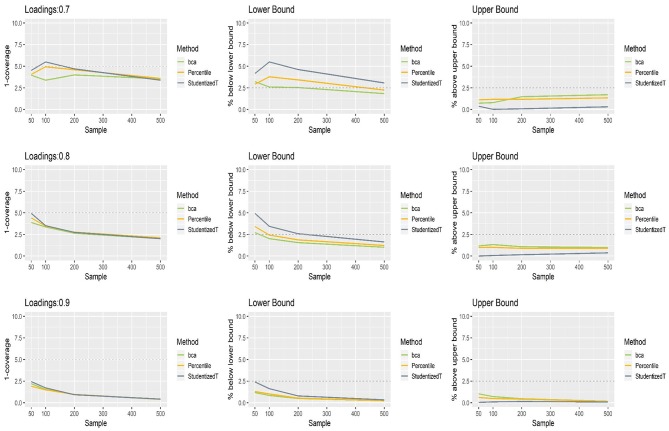
The coverage and balance of percentile, bias-corrected and accelerated bootstrap (BCa), and Student's *t* 95% confidence intervals (CIs) of 0.7, 0.8, and 0.9 loadings for normal indicators.

Figure 2 shows the coverage and balance of the percentile, BCa, and Student's *t* 95% CIs of the loadings ranging from 0.7 to 0.9 under the normal distributions. In the case of 0.9 loading, each method produced CIs that included this population value more than 95% of time, implying conservative wider coverage with a less than nominal level of 5% error rate. The same results were found in the 0.8 loading condition, except for a small sample (*N* = 50). When loading was 0.7, Student's *t* CIs excluded this population value more than 5% of the time if they were constructed using a small sample (*N* = 100). For a larger sample (i.e., >200), overall, each of the percentile, BCa, and Student's *t* CIs was farther away from the desired level of coverage (lower than the horizontal dashed line at 5%), indicating wider CIs of $\hat{\theta }$. This tendency becomes greater as the loading size gets larger. In general, the percentile method produced better coverage in CIs (i.e., closer to the horizontal dashed line) than did the other two methods.

All three bootstrap methods tended to shift their CIs upward, and such shifting was problematic particularly with smaller loadings (0.7, 0.8) and smaller sample sizes (*N* = 50, 100). Consequently, the CIs were imbalanced such that the lower limit was greater than the population value more often than would be desired (2.5%), while the upper limit was smaller than the population value less frequently than would be desired (2.5%). For the loading of 0.7, the balance in CIs improved as sample size increased (i.e., closer to the nominal level of 2.5%). Under the conditions of 0.9 loading and the sample size > 100, the population value was smaller than the lower limit or greater than the upper limit fewer times than would be desired primarily due to the inflated coverage (i.e., >95%) of wide CIs. Overall, imbalanced and inflated coverage was observed across all the methods.

### Loadings for Non-normally Distributed Indicators

**Figure 3:**
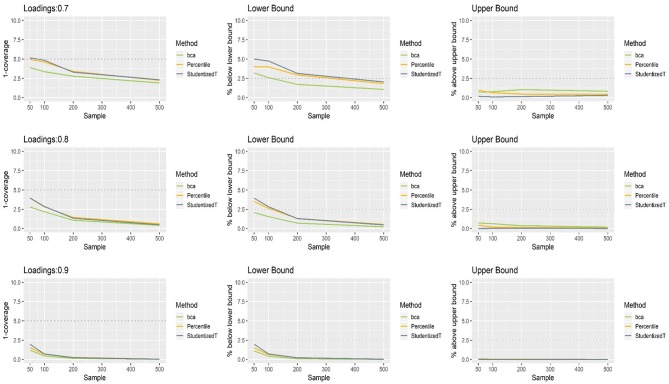
The coverage and balance of percentile, bias-corrected and accelerated bootstrap (BCa), and Student's *t* 95% confidence intervals (CIs) of 0.7, 0.8, and 0.9 loadings for non-normally distributed indicators.

Figure 3 presents the coverage and balance of percentile, BCa, and Student's *t* 95% CIs of 0.7, 0.8, and 0.9 loadings for non-normally distributed indicators. The results were similar to those with normally distributed indicators, suggesting that the distribution of indicators has little impact on the bootstrap CIs of a loading. More than 95% of the time, the population value was included within the CIs regardless of loading size, and this proportion became greater as sample size increased. In general, coverage was comparable across the three methods, although the percentile and Student's *t* CIs were closer to the nominal level than the BCa CIs in the case of smaller loadings (0.7, 0.8). In general, the percentile method produced better coverage in CIs than did the other two methods.

The CIs of each method were shifted upward especially when relatively small loadings (0.7, 0.8) were estimated with a small sample (*N* = 50, 100). Under the conditions of 0.7 loading and larger sample sizes (*N* = 200, 500), the lower limit of the CIs was close to the theoretical coverage range—if compared, much closer to the desired level (2.5%) in the percentile and Student's *t* methods. For 0.9 loading, the CIs were wide (i.e., inflated coverage approaching 100%) so that the population value was smaller than the lower limit or greater than the upper limit many fewer times than would be desired (2.5%). Overall, the balance in CIs was not considerably different between the three methods.

### Path Coefficients With Normally Distributed Indicators

The simulation results for path coefficients were more similar across different loading values (0.7, 0.8, 0.9), suggesting that loading size has minimal influence on the bootstrap CIs. Thus, the results were aggregated over the three conditions of population loading, and the aggregated results are reported here. A full scope of the simulation results will be available upon request.

**Figure 4:**
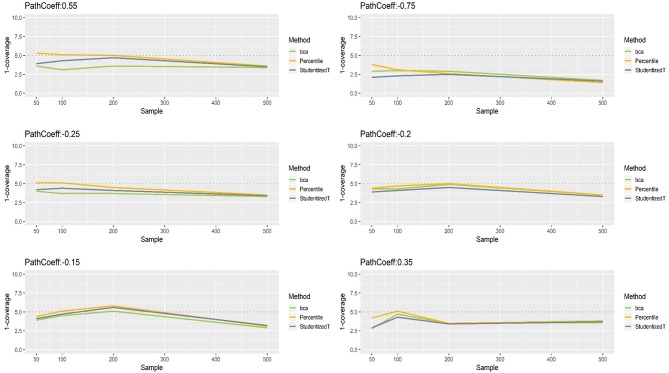
The coverage of percentile, bias-corrected and accelerated bootstrap (BCa), and Student's *t* 95% confidence intervals (CIs) of path coefficients in the normal indicator condition.

Figure 4 presents the coverage of percentile, BCa, and Student's *t* 95% CIs of path coefficients in varying size (−0.75, −0.25, −0.20, −0.15, 0.35, 0.55). First, the percentile method produced clearly better coverage in CIs (i.e., closer to the horizontal dashed line) than did the other two methods, regardless of the size of path coefficients but except for the largest path coefficient (−0.75). Second, the BCa and Student's *t* methods produced wider CIs than the desired level in coverage, and this tendency was more apparent as the absolute value of path coefficient increased. Third, the CIs became wider (i.e., inflated coverage) as sample size increased. In general, the percentile CIs were closer to the theoretical coverage range as compared to the other two methods.

**Figure 5:**
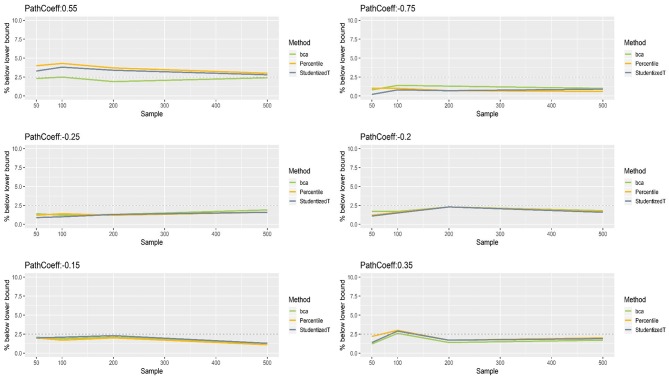
The lower limits of percentile, bias-corrected and accelerated bootstrap (BCa), and Student's *t* 95% confidence intervals (CIs) of path coefficients in the normal indicator condition.

**Figure 6:**
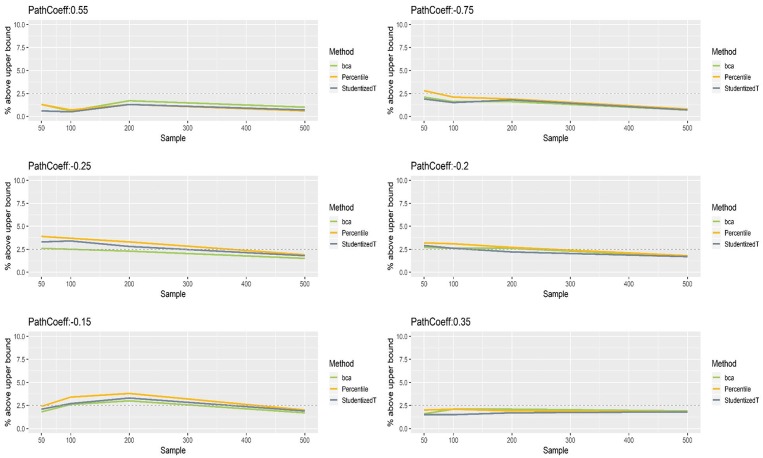
The upper limits of percentile, bias-corrected and accelerated bootstrap (BCa), and Student's *t* 95% confidence intervals (CIs) of path coefficients in the normal indicator condition.

Figures 5, 6 show that the CIs of small to moderate path coefficients (−0.25, −0.20, −0.15, 0.35) method were reasonably well-balanced—i.e., the lower and upper limits were close to the desired level (2.5%). In contrast, for relatively large path coefficients (−0.75, 0.55), the population value was smaller than the lower or greater than the upper limit many fewer times than would be desired. Overall, the BCa method was less prone to imbalance than the percentile and Student's *t* methods.

### Path Coefficients With Non-normally Distributed Indicators

**Figure 7:**
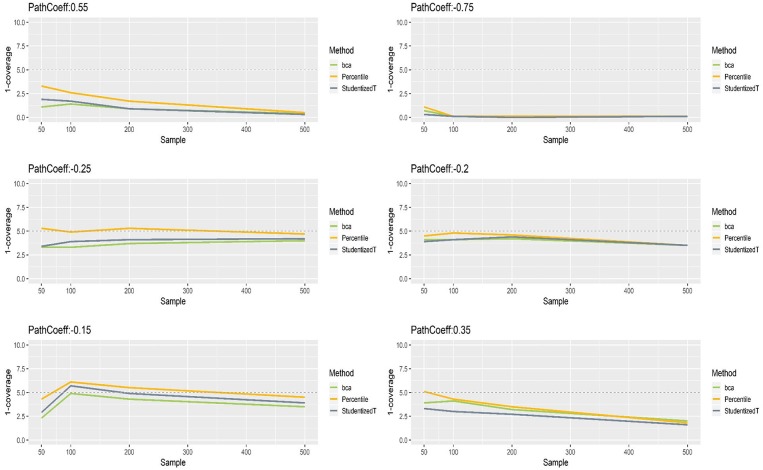
The coverage of percentile, BCa, and Student's *t* 95% CIs of path coefficients in the non-normally distributed indicator condition.

Figure 7 presents the coverage of percentile, BCa, and Student's *t* 95% CIs of path coefficients when the indicators were non-normally distributed. The results were somewhat different from what we found with normally distributed indicators. Specifically, the CIs of each method were still close to the theoretical coverage range (95%) when the population value of path coefficients was relatively small to moderate (−0.25, −0.20, −0.15), whereas the CIs that included the population value of large path coefficients (−0.75, 0.35, 0.55) had much wider CIs (i.e., much more than 95% of time). Such inflation in coverage worsened as sample size increased. For instance, when path coefficient was −0.75 and sample size was >100, all three methods produced CIs that included the population value almost always over replications. Nevertheless, overall, the percentile CIs were less sensitive to the population value and sample size, showing closer to the nominal level of 95%, compared to the other two methods.

**Figure 8:**
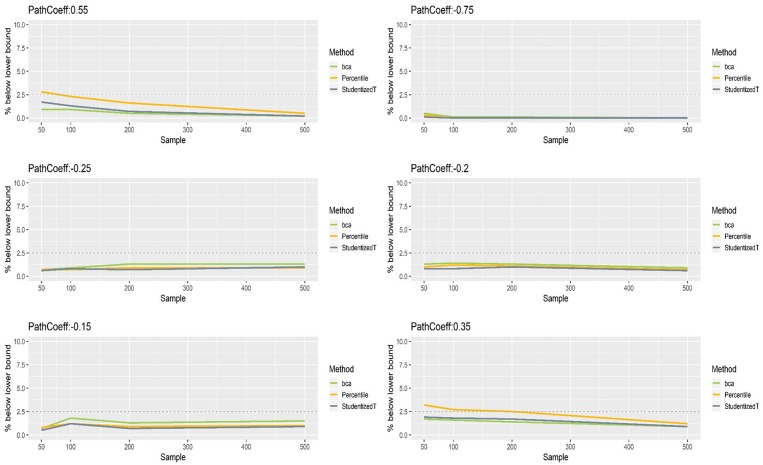
The lower limits of percentile, bias-corrected and accelerated bootstrap (BCa), and Student's *t* 95% confidence intervals (CIs) of path coefficients in the non-normally distributed indicator condition.

**Figure 9:**
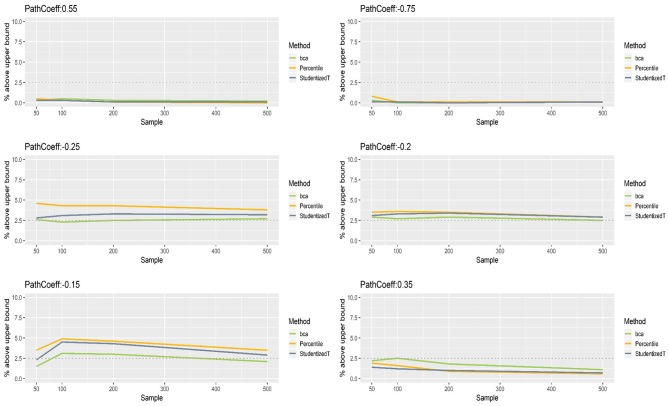
The upper limits of percentile, bias-corrected and accelerated bootstrap (BCa), and Student's *t* 95% confidence intervals (CIs) of path coefficients in the non-normally distributed indicator condition.

Figures 8, 9 show that the CIs of small to moderate path coefficients (−0.25, −0.20, −0.15) were shifted slightly downward—i.e., the lower limit was lower than the desired level (2.5%), and the upper limit was higher than the desired level (2.5%). The balance somewhat improved as sample size increased (i.e., closer to the nominal level of 2.5%). In contrast, for relatively large path coefficients (−0.75, 0.55), the population value was smaller than the lower limit or greater than the upper limit many fewer times than would be desired, probably due to the inflated coverage (i.e., wide CI) observed earlier. Similar to the findings in the normal indicator condition, the BCa method was less prone to imbalance than the percentile and Student's *t* methods.

## Discussion

The present study successfully implemented percentile, BCa, and Student's *t* CI methods into GSCA, further exploring the capability of statistical inference with GSCA, and investigated the performance of the different bootstrap methods in terms of coverage and balance of their CIs. A number of important findings emerged from our simulation work. First, the distribution of indicators had little impact on the bootstrap CIs of a loading. Second, all three methods produced wider CIs that included the population loading value more frequently than would be desired. Such inflation in coverage was greater when the CIs were constructed with a larger sample. Third, the CIs of a loading were shifted upward, causing imbalance of CIs. Fourth, the size of indicator loadings did little to affect the bootstrap CIs of a path coefficient, but the distribution of indicators had substantial impact, yielding fairly different performances of the CIs. When the indicators were normally distributed, the percentile CIs produced closer to the theoretical coverage range as compared to BCa and Student's *t* CIs, while the CIs of the BCa method were better balanced than the other two methods. When the indicators were non-normal rather than normal, the CIs of each method included the population value of large path coefficients more often than would be desired (i.e., inflated coverage), while the CIs of small path coefficients showed ideal levels of coverage and balance. Lastly, the coverage of percentile CIs was less sensitive to the population value and sample size than BCa CIs and Student's *t* CIs, while the BCa method was less prone to imbalance than the other two methods.

The current study findings have important implications for researchers in substantive areas of statistical inferences using bootstrap CIs in GSCA. The choice over different CI methods should be carefully considered, especially when the sample size is small (e.g., 50 or 100). Our stimulation results revealed an outperformance of the percentile method over both BCa and Student's *t* with respect to the desired coverage of the CIs. The BCa method tended to produce slightly better-balanced CIs than the other two methods, but overall, it was undermined due to its narrower CIs than the desired level in coverage. For empirical research, the computational efficiency of the three methods would also be considered. The percentile method is more efficient in computation speed compared to the BCa method because the latter has additional calculation steps for the parameters of acceleration and bias correction. An efficient algorithm is desired especially in situations where solutions have to be obtained repeatedly, like the current simulation study. Thus, we would recommend the adoption of percentile method as a standard procedure for GSCA due to its better CI coverage at the nominal level and its computational efficiency.

Future studies would expand the current simulation scope to other GSCA estimation methods and various advanced GSCA models for a comparison among different CI methods. One direction for future studies is to compare the different bootstrap CI methods in a regularized extension of GSCA (rGSCA; Hwang, 2009), which combines a ridge type of regularization into GSCA in a unified framework, thereby handling potential multicollinearity problems more effectively. In a simulation study, rGSCA was found to provide parameter estimates that are as good as or better than those from original GSCA in various conditions of normally distributed data. Furthermore, we may also consider a simulation study for comparisons of the CI methods in GSCA with uniqueness terms for accommodating measurement error (GSCA_M_; Hwang et al., 2017), which has been proposed to extend the original GSCA to account for errors in indicators explicitly. This extension contemplates both common and unique parts of indicators and estimates a weighted composite of indicators with their unique parts removed. Unlike the original GSCA, GSCA_M_ has a bias-correction method, dealing with measurement errors in indicators. Therefore, it would be warranted to consider a Monte Carlo simulation on the relative performance of the different CI methods with GSCA_M_ as well as rGSCA.

Another direction for future studies is to compare the different CI methods in a broad range of conditions and models for more rigorous investigations. In particular, it would be necessary to examine the relative performance of each CI method with variant GSCA models such as fuzzy clusterwise GSCA for handling cluster-level respondent heterogeneity, multilevel GSCA, GSCA with latent interactions, and dynamic and functional GSCA for longitudinal data and time series data (Hwang and Takane, 2014). The current study focused on 95% confidence level and sample sizes ≤ 500 because they are most frequently encountered in practice. Future research on the use of different confidence levels and larger samples (e.g., 1,000–3,000) is also warranted to provide more practical implications for applied research. These rigorous investigations in the advanced modeling framework would provide applied researchers with information on which CI method would be better across different experimental conditions and models. This would ascertain the relative benefits of each CI method.

## Data Availability Statement

The MATLAB codes for data generation are available in Supplementary Material.

## Author Contributions

KJ, JL, and VG contributed to technical development, empirical analyses, and manuscript writing. GC contributed to technical development and manuscript writing.

### Conflict of Interest

The authors declare that the research was conducted in the absence of any commercial or financial relationships that could be construed as a potential conflict of interest.

## Footnotes

^1^Unlike Cho et al. (2019), structural error terms were assumed to be independent of each other for simplicity.

^2^We interchangeably used the terms latent variables and composites/components because, in GSCA, a latent variable is defined as a weighted composite or component of observed variables.
