# Supplementary material for: Comparison of Bootstrap Confidence Interval Methods for GSCA Using a Monte Carlo Simulation
Source: Front Psychol. 2019 Oct 11;10:2215. doi: 10.3389/fpsyg.2019.02215 (PMC6797821; doi:10.3389/fpsyg.2019.02215)
Supplement: Supplementary file 1 [file Data_Sheet_1.PDF]

```
%%  Data generating code for Jung, Lee, Gupta, & Cho (2019)
%  - This script is based on the code developed and used for Cho, Jung, & Hwang (2019).
%  - The external function, DGP_CBSEM(), used in this script can be downloaded at the following link:
%      "https://www.researchgate.net/publication/335402351_DGP_function_described_in_Appendix"
%  - The last revision date is 2019/8/18

%% Step 1. Prescribe parameter values of W_ind, k, Bx, and Sig_Gx

%For loading values of 0.7
W_ind=[0.33 0.33 0.33]'; % unstandardized weight vector of indicators for each l.v.
k=0.235;                 % correlations among each block of indicators

%For loading values of 0.8
W_ind=[0.33 0.33 0.33]'; % unstandardized weight vector of indicators for each l.v.
k=0.460;                 % correlations among each block of indicators

%For loading values of 0.9
W_ind=[0.33 0.33 0.33]'; % unstandardized weight vector of indicators for each l.v.
k=0.715;                 % correlations among each block of indicators

Bx=[0.55 -0.75;      % path coefficients of exo l.v. relating to endo l.v
   -0.25 0;
    0   -0.20;
   -0.15 0.35];
r=0.5;               % correlation matrix for exogenous l.v.
Sig_Gx=[1 r -r  r ;
        r 1  0  0  ;
       -r 0  1  -r ;
        r 0  -r  1 ];

%% Step 2. Derive the covariance matrix
Sig_Z = DGP_CBSEM(1,W_ind,k,Bx,Sig_Gx);

%% Step 3. Generate data
Nrep=500;
N_vector=[50 100 200 500];
N_N=size(N_vector,2);
Z_final=cell(4,1);
type=1; % 1 = normal, 2 = log-normal
```

```
for n=1:N_N
    number_of_n=N_vector(n);
    [~,Z_final{n}] = DGP_CBSEM(2,[],[],[],[],Sig_Z,number_of_n,Nrep,type);
end
save generatedData.mat Z_final
```
