# Supplementary material for: Comparison of Bootstrap Confidence Interval Methods for GSCA Using a Monte Carlo Simulation
Source: Front Psychol. 2019 Oct 11;10:2215. doi: 10.3389/fpsyg.2019.02215 (PMC6797821; doi:10.3389/fpsyg.2019.02215)
Supplement: Supplementary file 2 [file Data_Sheet_2.PDF]

```
###########################################
##### R Functions for Three CIs      #####
###########################################

##Function to compute studentized t- interval (Sample size=50)
####################################################################
studTBoot_50 <- function(x,y){
  stdd <- apply(y,2,sd)
  tn <- qt(.975, df=49)
  tp <- stdd * tn
  tinterval <- cbind(x - tp,x + tp)
  result <- list(tinterval=tinterval)
  return(result)
}

##Function compute bounds for percentile method
#######################################################
lb <- ceiling(N_Boot*0.025)
ub <- ceiling(N_Boot*0.975)
CIPercentBounds <- function(x){

  for(i in 1:length(x)){

    if(ncol(x[[i]])==1){

      xx <- cbind(x[[i]][[lb]],x[[i]][[ub]])
      result <- list(xx=xx)
      cc1 <- rbind(cc1,result)
    }else{
      t <- matrix(0,ncol(x[[i]]),2)
      for(j in 1:ncol(x[[i]])){
        xx1 <- cbind(x[[i]][,j][lb],x[[i]][,j][ub])
        t[j,] <- xx1
        result1 <- list(t=t)
      }
      cc1 <- rbind(cc1,result1)
    }
  }
  return(cc1)
}




#####################################################
##confidence interval using BCa Method
####################################################

##Function to compute bias correct boot
##################################################
s <- c()
biasCorrectBoot <- function(x,y){
```

```
  if((length(x)!=0) && (length(y)!=0)){

    if(length(x)==1){

      w <- qnorm(sum(y<x)/nrow(y))
      s <- rbind(s,w)


    } else{

      for(i in 1:ncol(y)){
        t <- qnorm(sum(y[,i]<x[i,])/nrow(y))
        s <- rbind(s,t)
      }
    }
    result <- list(s=s)
    return(result)
  }
}

###Function to compute bias correction factor for all lists
################################################################
biasCorrectFactor <- function(x){

  i <- 1
  j <- 3

  biasF <- c()
  while(i<3 && j<5){

    bcaApply <- biasCorrectBoot(x[[i]],x[[j]])
    biasF <- c(biasF,bcaApply)
    i <- i+1
    j <- j+1

  }
  return(biasF)
}


###confidence interval using BCa Method
######################################################

  biasCorrectionFactor <- lapply(ss_bca[m], FUN=biasCorrectFactor)

  ##Jackknife sampling
  ########################################
  rrb <- seq(-1,-nrow(actualData))
  jse <-list()

  for(i in 1:length(rrb)){
    rr <- actualData[rrb[i],]
    jse[[i]] <- rr
```

```
}

theta <- list()
for(i in 1:length(jse)){

  ################################
  ## Run "gesca" r package here
  ################################

  }

meanTheta <- list()
sumTheta <- sumParam(length(theta[[1]]),theta)
meanTheta <- lapply(sumTheta,"/",length(theta))

names(meanTheta) <- names(theta[[1]])

###Calculating acceleration factor
################################################
diff <- theta
atop <- list(list())
abot <- list(list())

###Compute atop
############################
for(i in 1:length(theta)){

  for(j in 1:length(meanTheta)){

    diff[[i]][[j]] <- meanTheta[[j]] - theta[[i]][[j]]
    diff[[i]][[j]] <- diff[[i]][[j]]^3

  }
  atop[[i]] <- diff[[i]]
}
atop <- sumParam(length(atop[[1]]),atop)

###Compute abot
#############################################
diff <- theta
for(i in 1:length(theta)){

  for(j in 1:length(meanTheta)){

    diff[[i]][[j]] <- meanTheta[[j]] - theta[[i]][[j]]
    diff[[i]][[j]] <- diff[[i]][[j]]^2
  }
  abot[[i]]<- diff[[i]]
}
abot <- sumParam(length(abot[[1]]),abot)
abot <- lapply(abot,FUN= function(x) (x^1.5)*6)
```

```
###acceleration factor
########################################
accelerationFactor <- mapply("/",atop,abot,SIMPLIFY = FALSE)
accelerationFactor <- accelerationFactor[!is.na(accelerationFactor)]

###Confidence Interval
#########################################

alpha1 <- list()
alpha2 <- list()
for(j in 1:length(accelerationFactor)){

  x <- biasCorrectionFactor[[1]][[j]]

  alpha1[[j]] <- pnorm(x + ((x+qnorm(.025))/(1- (accelerationFactor[[j]]*(x+qnorm(.025))))))
  alpha2[[j]] <- pnorm(x + ((x+qnorm(.975))/(1- (accelerationFactor[[j]]*(x+qnorm(.975))))))

}

alpha <- list()
for(i in 1:length(alpha1)){

  lb <- ceiling(1000*alpha1[[i]])
  ub <- ceiling(1000*alpha2[[i]])
  alpha[[i]]<- list(lb=lb,ub=ub)
}
```
